# Raman micro-spectroscopy for accurate identification of primary human bronchial epithelial cells

**DOI:** 10.1038/s41598-018-30407-8

**Published:** 2018-08-22

**Authors:** Jakub M. Surmacki, Benjamin J. Woodhams, Alexandria Haslehurst, Bruce A. J. Ponder, Sarah E. Bohndiek

**Affiliations:** 1 Department of Physics, University of Cambridge, Cavendish Laboratory, JJ Thomson Avenue, Cambridge, CB3 0HE United Kingdom; 2 Cancer Research UK Cambridge Institute, University of Cambridge, Li Ka Shing Centre, Robinson Way, Cambridge, CB2 0RE United Kingdom

## Abstract

Live cell Raman micro-spectroscopy is emerging as a promising bioanalytical technique for label-free discrimination of a range of different cell types (e.g. cancer cells and fibroblasts) and behaviors (e.g. apoptosis). The aim of this study was to determine whether confocal Raman micro-spectroscopy shows sufficient sensitivity and specificity for identification of primary human bronchial epithelial cells (HBECs) to be used for live cell biological studies *in vitro*. We first compared cell preparation substrates and media, considering their influence on lung cell proliferation and Raman spectra, as well as methods for data acquisition, using different wavelengths (488 nm, 785 nm) and scan protocols (line, area). Evaluating these parameters using human lung cancer (A549) and fibroblast (MRC5) cell lines confirmed that line-scan data acquisition at 785 nm using complete cell media on a quartz substrate gave optimal performance. We then applied our protocol to acquisition of data from primary human bronchial epithelial cells (HBEC) derived from three independent sources, revealing an average sensitivity for different cell types of 96.3% and specificity of 95.2%. These results suggest that Raman micro-spectroscopy is suitable for delineating primary HBEC cell cultures, which in future could be used for identifying different lung cell types within co-cultures and studying the process of early carcinogenesis in lung cell culture.

## Introduction

Raman spectroscopy is a powerful bioanalytical technique that reveals the chemical constituents of a given sample based on the inelastic scattering properties of molecular bonds. Despite the relatively weak nature of the Raman effect (fewer than 1 Raman scattering event occurs for every 10^7^ elastic scattering events)^1^, the advent of confocal Raman micro-spectroscopy methods that allow 3D localization of signals together with highly sensitive detectors have enabled this label-free technique to be applied in living cells over the past decade, as extensively reviewed^2–8^. In particular, the ability to monitor the concentration of lipids, proteins and nucleic acids enables interrogation of a wide range of cellular processes. Examples range from identification and spatial localization of the main cellular components^9–12^, through time lapse studies of live and apoptotic cells^13–15^, to discrimination of normal and cancer cells^16–20^.

The aim of this study was to determine whether confocal Raman micro-spectroscopy would show sufficient sensitivity and specificity for identification of primary human bronchial epithelial cells (HBECs) as well as immortalized cell lines to be used for live cell biological studies *in vitro*. Despite the apparent promise for Raman spectroscopy in this application^8^, several challenges must be overcome to apply Raman spectroscopy in live cell studies, which have also been identified by recent reviews^2,4,6^. For example, the low probability of Raman scattering leads to a direct trade-off for live cell imaging between high signal-to-noise ratio, requiring long acquisition times, and adequate temporal resolution, required for longitudinal imaging of biological dynamics in live cells. Therefore, to achieve our aim, we first established a detailed protocol for imaging of live human lung cells *in vitro* by directly comparing methods for sample preparation and data acquisition. We performed these initial optimization studies in live human lung cancer (A549) and fibroblast (MRC5) immortalized cell lines and compared the imaging results qualitatively with fluorescence imaging. We then applied the optimized protocol to acquire data from primary HBECs from several different sources. Using partial least squares discriminant analysis, we achieved an average sensitivity of 96.3% and specificity of 95.2%, suggesting that Raman micro-spectroscopy may indeed be suitable for differentiating between HBEC primary cell cultures and could in future be applied to identification of different lung cell types within co-cultures and studying the process of early lung carcinogenesis in cell culture.

## Results

### Comparison of cell preparation and data acquisition methods for delineating cancer and fibroblast cell lines

Firstly, we evaluated the impact of different cell preparation conditions. Raman spectroscopy of cell substrates and culture media was performed at 488 nm and 785 nm (Supplementary Fig. 1). These results indicated that, in line with previous work^21^, a quartz substrate provides the best compromise for live lung cell imaging. In addition to the expected strong Raman peaks due to water at around 1640, 3250 and 3430 cm^−1^, cell culture media contributes additional peaks at around 1046, 1305 and 1454 cm^−1^, however, compared to physiological buffered solutions (HBSS, LCIS and PBS) it does not have a detrimental impact on the proliferation of the cell cultures over extended time periods (up to 48 hours).

**Figure 1:**
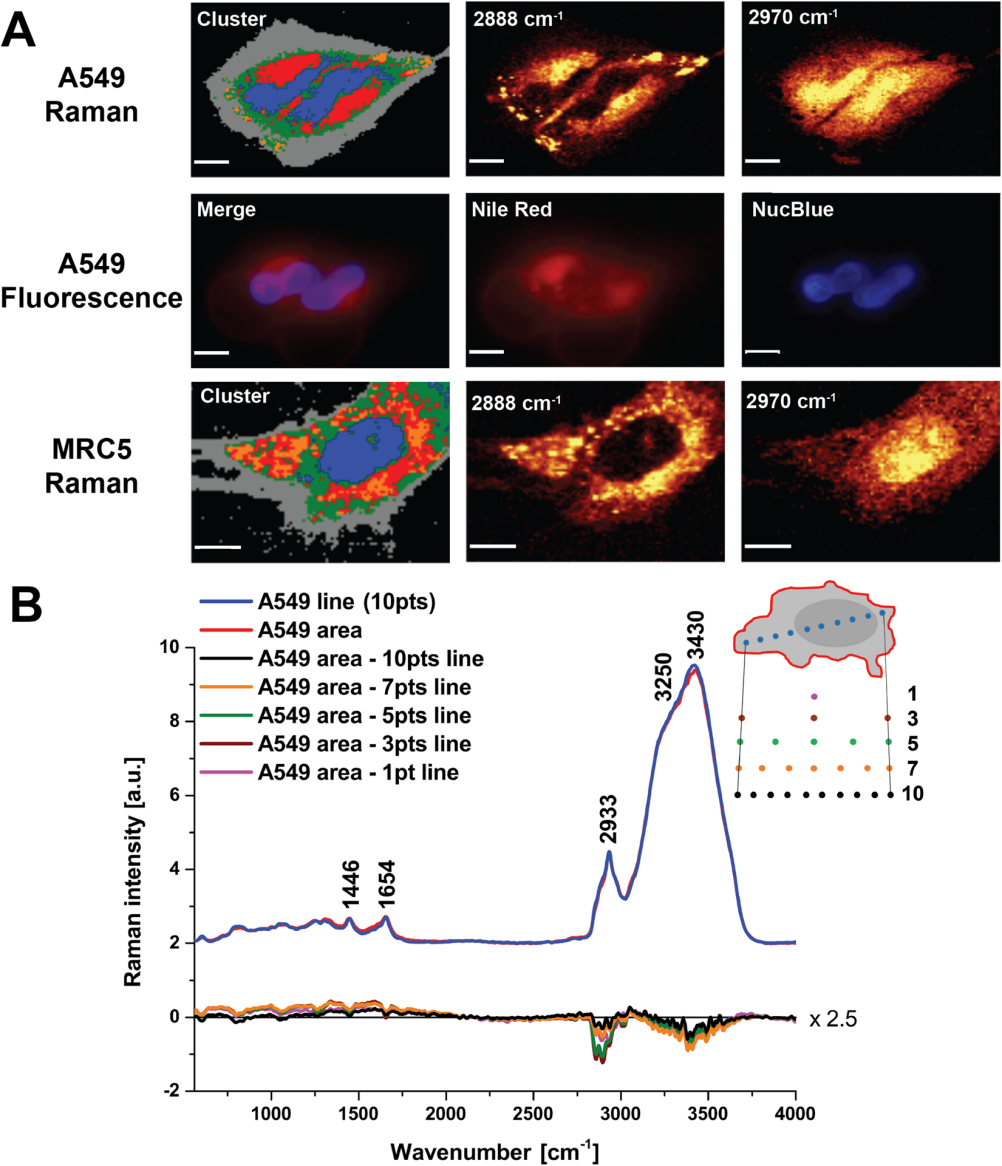
Comparison of area and line scan data acquisition from A549 and MRC5 cells. (**A)** Area scan Raman and fluorescence imaging data at 488 nm. Clusters were derived using Manhattan analysis (pre-mode: derivative). Cluster analysis reveals the following assignments based on spectra presented in Supplementary Figure 2: Black (cluster 1) = area without the cells (background); Grey (cluster 2) = cell border; Green (cluster 3) = cytoplasm; Blue (cluster 4) = nucleus; Red (cluster 5) = endoplasmic reticulum/mitochondria; Orange (cluster 6) = lipid droplets. For comparison, the lipid distribution at 2888 cm^−1^ (sum filter: 2838–2938 cm^−1^) is shown relative to fluorescence Nile Red staining in A549, while the nucleus area represented by 2970 cm^−1^ (sum filter: 2920–3020 cm^−1^) is compared to NucBlue. Raman area scans of A549: scale bar is 10 μm (148 × 100 points, 0.1 s per pixel, ~25 min per image); MRC5: scale bar is 9 μm (100 × 110 points, 0.1 s per pixel, ~20 min per image). **(B)** Comparing average of single Raman spectra along a line passing through the center of the cell (blue) to the full cell area scan (red) from A549 provides very similar results at 10 spectral samples, as shown in the differential spectrum (black). Average spectra were normalized to area under curve for this comparison.

Secondly, we compared results obtained using different data acquisition methods. Photothermal and photochemical reactions to laser illumination can rapidly induce cell death^22^. To avoid extended dwell time and allow more frequent Raman spectroscopy data acquisitions (technical replicates) from more cells (biological replicates) when studying primary HBECs, we examined the potential of using a line-scan rather than an area-scan data acquisition. We started by performing area-scans of lung A549 cancer cells and MRC5 fibroblast cells at 488 nm excitation using both K-means clustering and sum filters to generate Raman images (Fig. 1A). The associated cluster spectra are presented in Supplementary Figure 2 after background (cluster 1) subtraction. Epi-fluorescent imaging of the same A549 cell stained with NucBlue (nucleus) and Nile Red (lipids) after the Raman experiment are also shown in Fig. 1, which allowed us to perform a qualitative comparison of the lipid rich regions and nuclei location as described below. As the MRC5 cells are migratory, fluorescence staining and comparison could not be performed due to live cell motion.

The main differences observed between the clusters from the two cell types (examined in Supplementary Fig. 2) were in the cytoplasm (cluster 3), nucleus (cluster 4) and lipid droplet profile (cluster 6). In general, the spectra from the two immortalized cell lines indicate significant contributions from lipids, proteins and DNA/RNA components as expected from previous cell studies and reference spectra^23^. The most characteristic protein peaks observed arise from amides: amide A (NH stretching at around 3500 cm^−1^), amide B (NH stretching at around 3100 cm^−1^), and amides I to VII: amide I (1600–1690 cm^−1^ stretching vibration of C=O); amide II (1480–1580 cm^−1^ C-N stretching and N-H bending); amide III (1230–1350 cm^−1^ N-H/C-H deformation vibration modes); amide IV (625–770 cm^−1^ OCN bending); amide V (640–800 cm^−1^ NH bending); amide VI (540–600 cm^−1^ out of plane C=O bending); and amide VII (200 cm^−1^ skeletal mode)^24,25^. Characteristic fatty acid peaks could also be well distinguished using typical bands at around 1264, 1301 and 1440 cm^−1^ ^23^. Water vibrations in the high wavenumber region are assigned to: ~3250 cm^−1^ O-H symmetric stretch (weak shoulder) and ~3430 cm^−1^ O-H antisymmetric stretch (strong shoulder) weakly bound^26–28^. With decreasing ratio of water contribution from nucleus, cytoplasm to lipid droplets, we observed changes of the stretch peak intensities and positions, moving towards higher wavenumber (OH symmetric: from 3210 by 3220 to 3236 cm^−1^; OH antisymmetric: from 3458 by 3450 to 3430 cm^−1^).

**Figure 2:**
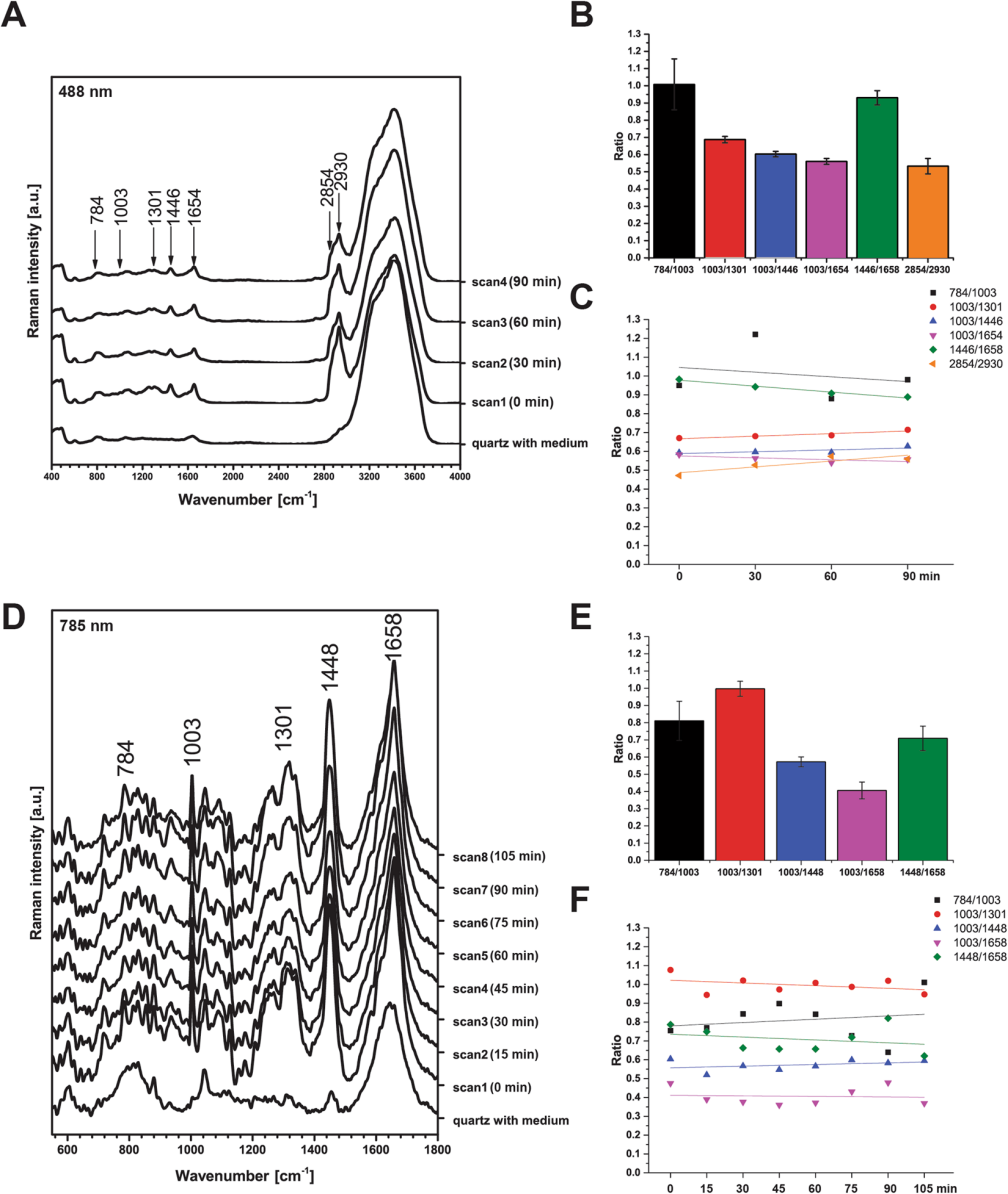
Impact of longitudinal line-scan data acquisition at multiple time points on live cell spectra. (**A**) Average Raman spectra of 10 points taken along a line across an A549 cell from scan 1 to scan 4 for 488 nm (10 mW, 0.5 s, 30 accumulations, 30 minutes between scans). Raman peak intensity ratios were calculated across all 4 scans at 488 nm (**B**) and also compared as a function of time (**C**) for: I(784/1003), pyrimidine bases, DNA/phenylalanine; I(1003/1301), phenylalanine/CH_2_ twist of lipids; I(1003/1446), phenylalanine/CH deformation; I(1003/1654), phenylalanine/mixed Amide I protein and C = C stretching of lipids; I(1446/1654), CH deformation/mixed Amide I protein and C = C stretching of lipids; and I(2854/2930) CH_2_ stretching of lipids/ CH_3_ symmetric stretching of proteins. (**D**) Average Raman line-scan spectra from scan 1 to 8 for 785 nm (120 mW, 1 s, 30 accumulations, 15 minutes between scans). Raman peak intensity ratios except for I(2854/2930) were calculated across all 8 scans at 785 nm (**E**) and also compared as a function of time (**F**). I(2854/2930) was inaccessible at 785 nm due to the installed diffraction grating. Fewer replicate scans were performed at 488 nm to avoid photodamage while enabling comparable time points to 785 nm. Spectra acquired using line-scan method.

Finally, having established the main Raman spectral features associated with different cellular components using these area-scans, we compared the average spectrum extracted from a line spanning a single cell (passing through the center) to the average spectrum from an area scan (Fig. 1B). We did this using different numbers of spectral samples along the length of the line, finding that 10 sample points was sufficient to recapitulate the average spectrum derived from the area scan while providing a significant increase in speed of data acquisition for live cells. To test whether the line scan acquisition had any impact on the biochemistry of the cells under study, we repeatedly acquired such line scans from the same individual cells over the course of 2 hours using 10 mW at 488 nm and 120 mW at 785 nm. At 488 nm excitation (Fig. 2A–C), no significant change is seen for I(784/1003), I(1003/1446), or I(1003/1654), however, trends are observed over 2 hours for I(1003/1301) (y = 0.014 ± 0.004x + 0.65 ± 0.01, R^2^ = 0.81), I(1446/1658) (y = −0.032 ± 0.003x + 1.01 ± 0.01, R^2^ = 0.97), and I(2854/2930) (y = 0.031 ± 0.01x + 0.46 ± 0.03, R^2^ = 0.67). Qualitative inspection and quantitative assessment of peak intensity ratios for data collected at 785 nm (Fig. 2D–F) were not found to change significantly over the repeated scans over a 2 hour time period for 785 nm, suggesting that 785 nm is relatively innocuous and would be preferable for repeated illumination of the same live cell^29^. For the remainder of our studies, we thus used 785 nm illumination to examine the fingerprint wavenumber region only.

### Analysis of live human immortalized lung cell lines

**Figure 3:**
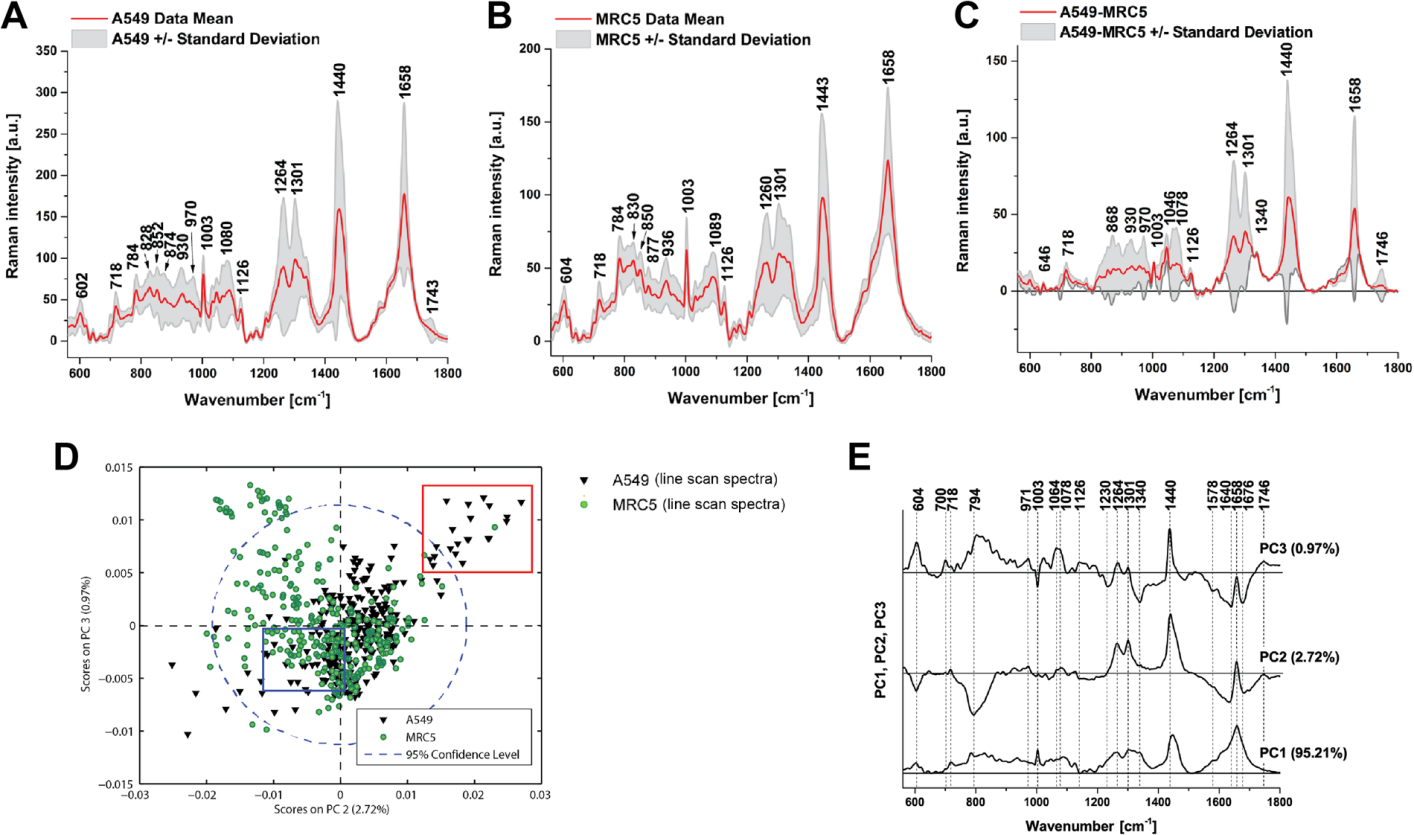
Comparison of spectral features of A549 and MRC5 immortalized cell lines. Mean and standard deviation (shaded area) of the non-normalized spectra recorded from 30 independent A549 (**A**) and MRC5 (**B**) cells are illustrated. Difference spectra are shown in (**C**) for direct comparison of the differences in molecular vibrations between the two cell lines. (**D**) Scatter plot of the score values of each single Raman spectrum for the second and third principal components (PCs) from the A549 (black triangles) and MRC5 cells (green circles). (**E**) Loadings plot of PC1, PC2 and PC3. Data acquired at 785 nm with 1 sec exposure and 30 accumulations per single spectrum. Total 300 spectra from 30 cells using the line-scan method for each cell line were analysed. Preprocessing PCA mode: normalization to area.

We first applied our live cell data acquisition protocol to compare immortalized human A549 cancer cells and MRC5 fibroblast cells to test the capability of the approach to distinguish different lung cell types. We performed replicate 10-point line-scan experiments over 30 cells of each cell type (300 spectra for each cell line), to establish the variability within and between cell lines using 785 nm laser excitation (Fig. 3A–C). The likely assignments to the observed peaks (based on the literature) are given in Supplementary Table 1. Comparing a total of 600 spectra from these cells, the variability within Raman spectra recorded across all replicates was sufficiently low as to enable detection of significant differences between these cancer and fibroblast cells (Supplementary Table 2) based on Raman bands from DNA (784 cm^−1^) and lipids (1264, 1301 and 1440 cm^−1^) as well as proteins (1003 and 1658 cm^−1^).

Principal components analysis (PCA) was then performed on the spectra of the MRC5 and A549 cell lines (Fig. 3D,E; PCA on second derivative spectra in Supplementary Fig. 3), and similar features were identified in the loadings plots in both cases. Based on this analysis, Raman spectra of lipid droplets determine a positive value of PC2 and PC3, while a negative value of PC2 and PC3 indicate spectral features arising from nucleus and cytoplasm. A549 cells show a higher value on PC3 at both wavelengths investigated, indicating the presence of lipid droplets in these cells is a distinguishing factor. Our PCA results indicate further subtle differences between A549 and MRC5 cell lines based on the Raman spectral profile of their lipid contributions (positive value of PC2 at 785 nm).

To analyze this further, we interrogated the spectra contributing to the different areas of the PCA scatterplots (Supplementary Fig. 4). Positive peaks on PC2 are at 1080, 1264, 1301, 1340, 1444, 1654, 2726, 2850, 2928 and 3056 cm^−1^ and on PC3 at 1424, 1633, 2842 and 2904 cm^−1^, while negative peaks on PC3 are at 1238, 1331, 1455, 1670, 2940, 2978 and 3064 cm^−1^. Negative values of PC3 could therefore describe mostly β-sheet protein. γ-globulin (mostly β-sheet proteins) has been found to be represented by bands at around 1239, 1334, 1450, 1671, 2940, 2975 and 3067 cm^−1^ ^30^.

### Identification of primary human bronchial epithelial cells in culture

**Figure 4:**
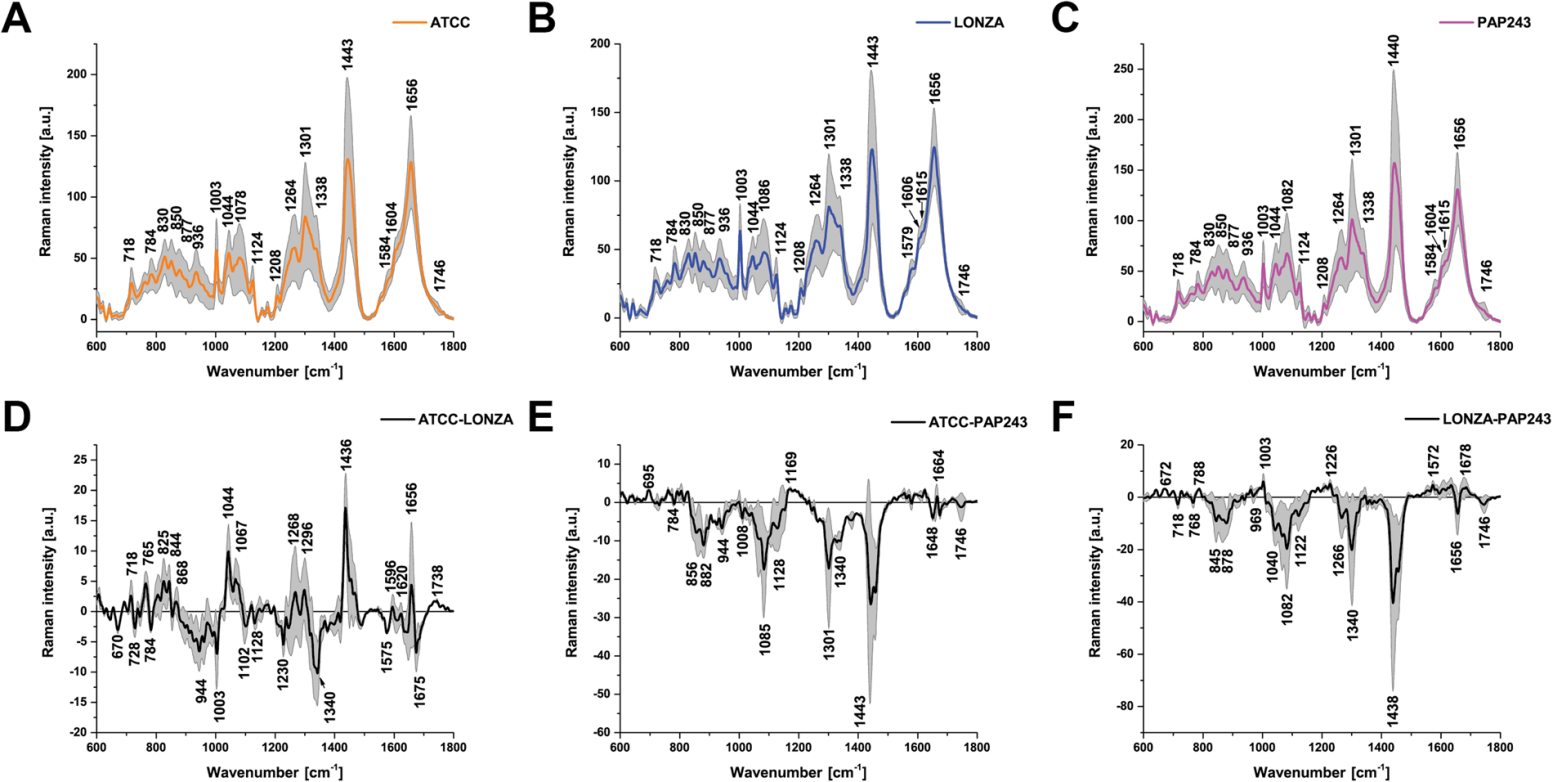
Comparison of spectral features of primary human bronchial epithelial cell (HBEC) lines. Mean and standard deviation (shaded area) of the non-normalized spectra recorded from 30 independent ATCC (**A**), LONZA (**B**) and PAP243 (**C**) cells are illustrated. Difference spectra are shown for direct comparison of the differences in molecular vibrations between ATCC and LONZA (**D**), ATCC and PAP243 (**E**) and LONZA and PAP243 (**F**). Data acquired at 785 nm with 1 sec exposure and 30 accumulations per single spectrum. Total 300 spectra from 30 cells using the line-scan method for each cell line were analysed.

**Figure 5:**
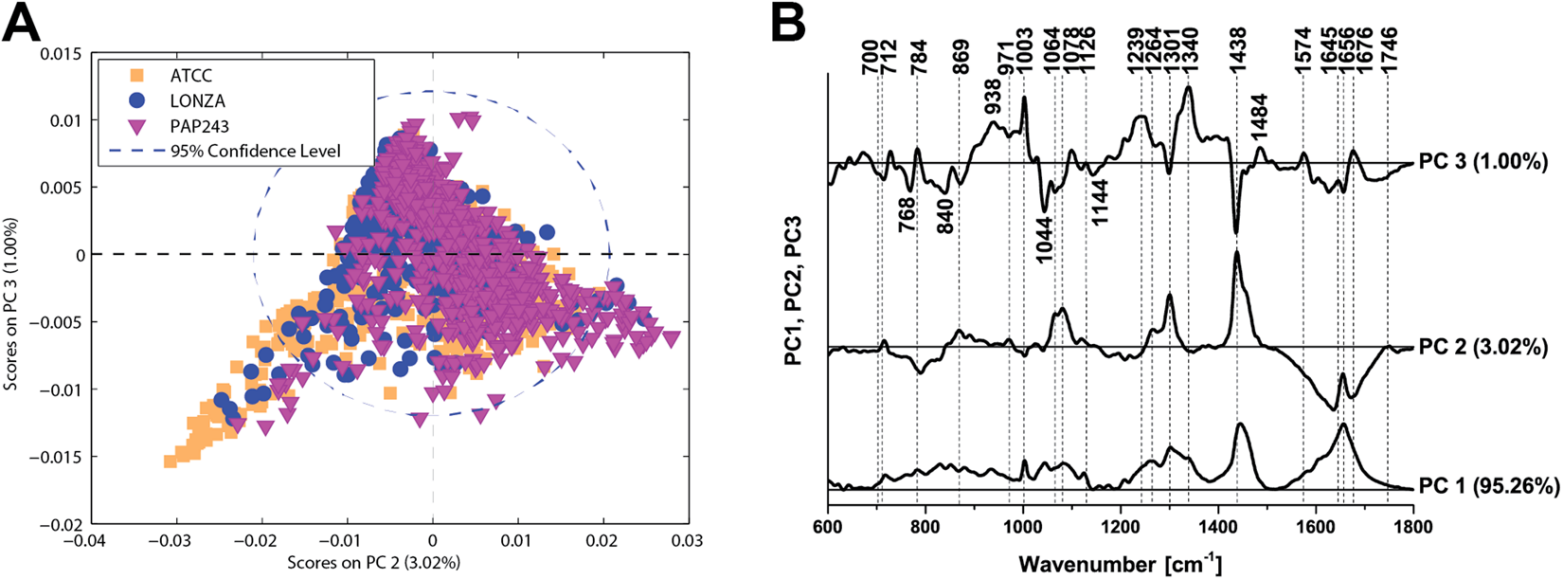
Principal components analysis of primary HBECs. (**A**) Scatter plot of the score values of each single Raman spectrum for the second and third principal components from the ATCC (orange squares), LONZA (blue circles), and PAP243 (purple triangles). (**B**) Loadings plot of PC1, PC2 and PC3. Data acquired at 785 nm with 1 sec exposure and 30 accumulations per single spectrum. Total 300 spectra from 30 cells using the line-scan method for each cell line were analysed. Preprocessing PCA mode: normalization to area.

Finally, we performed replicate 10-point line-scan experiments over 30 cells of each HBEC culture (300 spectra for each cell line) for three primary HBEC lines at 785 nm using our optimal protocol. To assess the ability to distinguish these HBEC cultures from one another we first repeated the analysis performed for the two immortalized cell lines, considering both mean spectra (Fig. 4A–C), difference spectra (Fig. 4D–F) and PCA (Fig. 5). Of particular value are the difference spectra, highlighting key spectral differences arising again from DNA (784 cm^−1^) and lipids (1264, 1301 and 1440 cm^−1^), proteins (1003 and 1658 cm^−1^) as well as a range of other peaks whose likely assignments (based on the literature) are given in Supplementary Table 1. PAP243, a primary HBEC line derived in-house, exhibited greater differences from each of the two commercial HBEC lines (Fig. 4E,F), than they did to each other (Fig. 4D). Perhaps highlighting the greater heterogeneity of the primary HBECs, a larger number of peaks contributed to the differences between the cell lines than with the immortalized cell line analysis. The primary HBECs are also more clearly separated using PCA (Fig. 5A), with negative values of PC2 and PC3 (Fig. 5B) providing delineation of ATCC and LONZA compared to PAP243.

**Figure 6:**
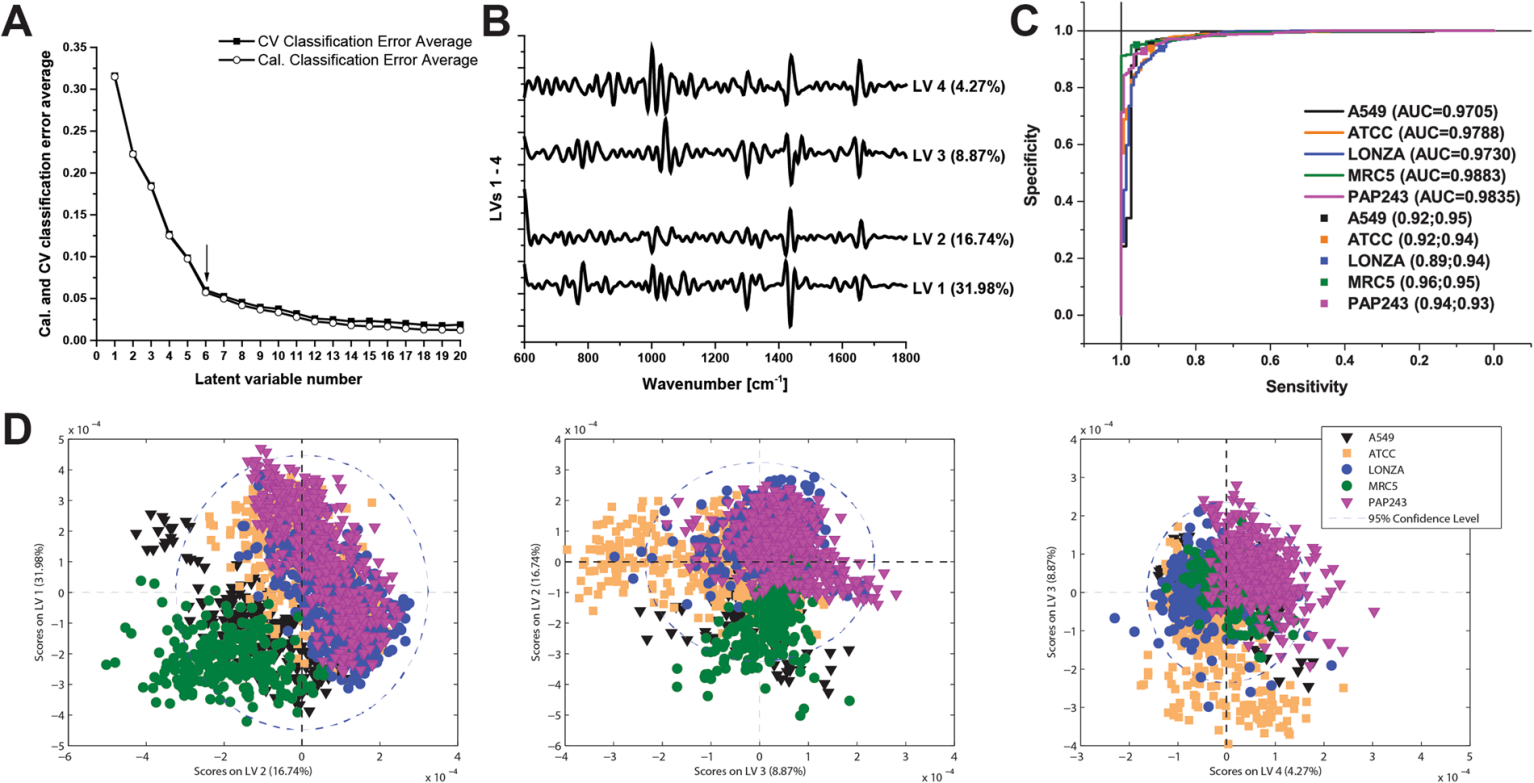
Partial least squares discriminant analysis (PLS-DA) of all Raman data MCR5, A549 and HBEC (ATCC, LONZA and PAP243). (**A**) Plot of calibration (Cal) and cross validation (CV) classification error average against the number of latent variables (LVs). (**B**) Plot of the loadings of LV1-LV4 against wavenumber. (**C**) Receiver operating characteristic (ROC) curves for the classification. (**D**) Scores plots on latent variables (LV1-LV4). Symbols: ATCC (orange squares); LONZA (blue circles); and PAP243 (purple triangles); A549 (black triangles); MRC5 (green circles). Preprocessing PLS-DA mode: vector normalization, 2^nd^ derivative, smoothing and mean center. The spectra were split into calibration and validation sets by removing every fourth spectrum to form the validation set. Spectra acquired using line-scan method.

To test the classification potential for the HBEC cultures, together with the immortalized cell lines, we performed partial least squares discriminant analysis (PLS-DA). PLS-DA is a chemometric technique widely used to optimize separation between different groups of samples^31–33^. We used 6 latent variables in our model (Fig. 6A) to set the calibration and cross validation classification error average at 5%. Loadings plots of the first 4 latent variables (LVs) and receiver operating characteristic (ROC) curves of all Raman data were also created (Fig. 6B,C). Areas under ROC curves were: A549 = 0.9705, ATCC = 0.9788, LONZA = 0.9730, MRC5 = 0.9883, PAP243 = 0.9835. Scores plots on LVs show good discrimination up to 4 LVs (Fig. 6D). The results of the classification of spectra into the 5 cell lines using all individual line scan spectra are shown in Table 1 (confusion matrix in Supplementary Table 3), with an average sensitivity of 92.4% achieved with an average specificity of 94.1%. Importantly, when analyzing the different biological replicates taken from each cell line separately, there was no significant difference observed between replicates of the same cell line. As further confirmation of performance, the root mean square error of calibration (RMSEC), cross-validation (RMSECV) and prediction (RMSEP) values were particularly low, confirming a good validity criteria for the calibration, cross-validation and prediction models formed using PLS-DA. We also calculated the classification results for the average line scan spectrum per cell (Table 2, confusion matrix in Supplementary Table 4). Interestingly, the classification of the average line-scan spectra showed improved performance, with an average sensitivity of 96.3% achieved with an average specificity of 95.2%, and lower RMSEC, RMSECV and RMSEP. This could be due to the fact that the average spectrum will contain contributions from most of the different subcellular components, whereas the individual spectra do not contain this complete picture.

**Table 1:** Classification results from the PLS-DA Analysis of all individual line-scan spectra.

Cell line	Sensitivity	Specificity	AUC	RMSEC	RMSECV	RMSEP
MRC5	96.0%	95.0%	0.9883	0.2472	0.2482	0.2569
A549	92.0%	94.9%	0.9705	0.2497	0.2518	0.2630
HBEC PCS-300–010 (LONZA)	88.7%	93.8%	0.9730	0.3749	0.3779	0.3679
HBEC CC-2440 (ATCC)	92.0%	94.0%	0.9788	0.3683	0.3707	0.3714
HBEC PAP243	93.3%	92.7%	0.9835	0.3625	0.3645	0.3555

Results include sensitivity, specificity, area under curve (AUC), root mean square error of calibration (RMSEC), cross-validation (RMSECV) and prediction (RMSEP) values for all cell lines (MRC5, A549 and HBECs).

**Table 2:** Classification results from the PLS-DA Analysis of all averaged line-scan spectra.

Cell line	Sensitivity	Specificity	AUC	RMSEC	RMSECV	RMSEP
MRC5	100%	98.2%	0.9897	0.1328	0.1462	0.1614
A549	100%	94.6%	0.9732	0.1352	0.1474	0.1631
HBEC PCS-300–010 (LONZA)	93.8%	91.7%	0.9648	0.2210	0.2450	0.2628
HBEC CC-2440 (ATCC)	93.8%	97.9%	0.9767	0.2064	0.2294	0.2138
HBEC PAP243	93.8%	93.8%	0.9831	0.2282	0.2454	0.2426

Results include sensitivity, specificity, area under curve (AUC), root mean square error of calibration (RMSEC), cross-validation (RMSECV) and prediction (RMSEP) values for all cell lines (MRC5, A549 and HBECs).

**Figure 7:**
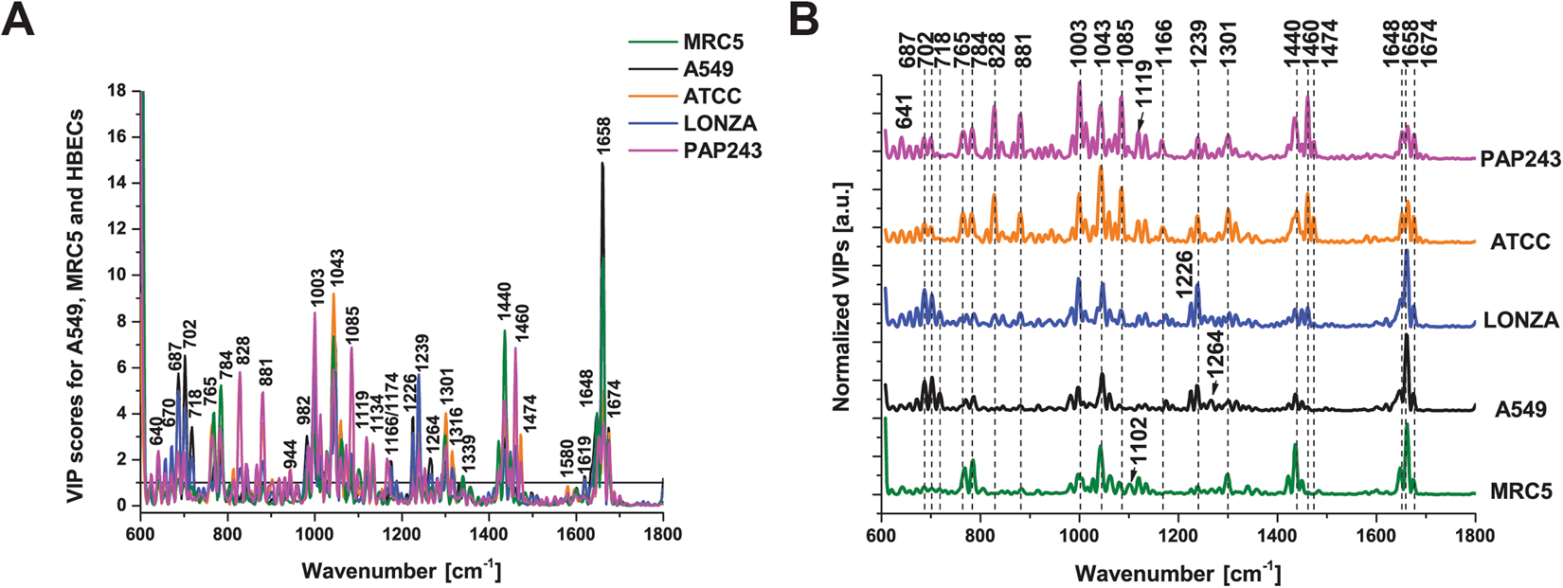
Variable Importance in Projection (VIP) scores for PLS-DA model of all Raman data. (**A**) VIP scores, shown offset for direct comparison in (**B**). Raman bands attributed to lipids (718, 1264, 1301, 1440 and 1658 cm^−1^), proteins (641, 1003, 1166/1174, 1239, 1580, 1658, 1674 cm^−1^), nucleic acids (784, 828, 1316, 1458 cm^−1^) and carbohydrates (881, 944, 1043, 1085 cm^−1^) can be observed to be the most important spectroscopic signatures for discrimination of the cell lines.

Variable importance in projection (VIP) scores greater than 1 help us to identify the spectral regions that are most important in providing optimal PLS-DA model performance^32^. The Raman bands that were most discriminatory in this study are highlighted in bold in Supplementary Table 1 and can be observed in Fig. 7A and offset in 7B. Raman bands attributed to lipids (718, 1264, 1301, 1440 and 1658 cm^−1^), proteins (641, 1003, 1166/1174, 1239, 1580, 1658, 1674 cm^−1^)^34,35^, nucleic acids (784, 828, 1316, 1458 cm^−1^)^34,35^ and carbohydrates (881, 944, 1043, 1085 cm^−1^)^35^ were found to be the most important spectroscopic signatures for discrimination of live cells of human lung origin and enable discrimination not only of immortalized cancer and fibroblast cells, but also of primary HBECs.

## Discussion

The aim of this study was to determine whether confocal Raman micro-spectroscopy would show sufficient sensitivity and specificity for identification of primary human bronchial epithelial cells (HBECs) as well as immortalized cell lines to be used for cell biological studies *in vitro*. Raman spectroscopy has shown promise for application in studies of living cells but given the subtle spectral changes that we wished to evaluate, it was crucial to first establish an optimal protocol for performing such studies in cells derived from the lung. We confirmed that using a quartz substrate in a commercial cell chamber contained within a microscope incubator and providing phenol-red free media containing serum yielded stable conditions for Raman spectroscopy data acquisition at 488 nm and 785 nm, but that the latter wavelength resulted in less perturbation of the cellular biochemistry. Our Raman spectral images of the cells built by k-means clustering were also in good correspondence with the fluorescence stained images of lipid and DNA distributions. Furthermore, we exploited a line scanning approach to maximize within a fixed time frame the number of technical replicates (Raman spectral acquisitions) and biological replicates (cells and samples interrogated) that could be performed.

Comparing data acquired from human lung cancer and fibroblast cell lines indicates that the Raman spectral features associated with lipids (bands at 1264, 1301, 1440 cm^−1^) might prove to be potentially useful biomarkers for monitoring the progression of carcinogenesis, in line with previous studies^16,20^. We also found statistically significant differences in the intensity of the Raman band attributed to DNA (784 cm^−1^). Barkur *et al*.^17^ showed similar results with rat C6 glioma and human SK-N-SH neuroblastoma cells. Additionally, Terentis *et al*.^16^ found that metastatic melanoma cells could be differentiated from normal skin fibroblast cells based on a higher level of nucleic acids (bands at 783, 812, 1101, 1319, 1481 and 1571 cm^−1^) in the nucleoli of normal cells and higher levels of lipid (718, 1126, 1300, 1436 and 1659 cm^−1^) and proteins (1003, 1246, 1340, 1451 and 1665 cm^−1^) in the cytoplasm of metastatic melanoma cells. Characteristic Raman bands of lipids and nucleic acids may therefore prove valuable in delineating human lung cancer and fibroblasts in live cell studies.

These results were then further underpinned by our ability to distinguish different cultures of primary human bronchial epithelial cells from each other and from the immortalized cells with high sensitivity (89–100%) and specificity (92–98%). In our PLS-DA studies, Raman bands attributed to lipids and DNA were again of importance, but additional bands relating to proteins and carbohydrates were also highly relevant. Using averaged line-scan spectra gave improved classification performance compared to individual line-scan spectra. These results suggest that Raman micro-spectroscopy may be suitable for identifying different primary HBEC cell cultures, which in future could be applied to identify different lung cell types within co-cultures and to study the process of early carcinogenesis in lung cell culture, of particular interest in the field of cancer research. Further refinements may also enable application in three dimensional lung cultures, which are increasingly being used in basic cell biology studies as they have been recognized to better recapitulate the physiological conditions and environments a cell experiences *in vivo*^36,37^.

While we have achieved reproducible measurements that allowed us to distinguish between the five live human lung cell types under test, there remain several limitations to this study. We anticipate that given the high degree of biological variation between cell lines, further tuning of the parameters identified here for optimal live lung cell Raman micro-spectroscopy may be necessary if the method is applied in other primary cell lines. Our findings should therefore be taken to represent a starting point for further study of HBECs in cell co-cultures or three-dimensional cell cultures. In addition, we restricted ourselves in this work to considering only spontaneous Raman spectroscopy; future studies should investigate the possibility for extending this approach into resonance and coherent Raman approaches to assist with multiplexing the measurement of additional biological processes^38–41^.

## Conclusions

Here, we developed and applied an optimized protocol for confocal Raman micro-spectroscopy to enable identification of different living human lung cell lines in culture. We used immortalized human cell lines derived from lung cancer (A549) and fibroblasts (MRC5) as well as three primary human bronchial epithelial cell (HBEC) lines. We were able to successfully delineate the immortalized cell lines based mainly on differences in lipid composition (Raman bands at 1264, 1301 and 1440 cm^−1^), particularly in the lipid droplets, and also based on differences in DNA content (784 cm^−1^). Those Raman bands were also important for optimal PLS-DA model performance of all 5 cell types including HBECs, where Raman bands attributed to lipids (718, 1264, 1301, 1440 and 1658 cm^−1^), proteins (641, 1003, 1166/1174, 1239, 1580, 1658, 1674 cm^−1^), nucleic acids (784, 828, 1316, 1458 cm^−1^) and carbohydrates (881, 944, 1043, 1085 cm^−1^) were of high relevance. The resulting PLS-DA model provided a high average sensitivity of 96.3% and specificity of 95.2%. Our approach could assist with the more widespread application of Raman spectroscopy in basic cell biology studies.

## Materials and Methods

### Micro-Raman spectroscopy and data analysis

Raman spectra were recorded on a confocal inverted Raman microscope (WITec Alpha 300 M+, WITec). The microscope is shown schematically in Supplementary Figure 5A. It is equipped with: a 488-nm diode laser (WITec); a 785 nm diode laser (XTRA II, Toptica Photonics); an Acton SpectraPro SP-2300, 300 mm triple grating imaging spectrometer (Princeton Instruments Inc.) with two 600 g/mm gratings (blazed at 500 and 750 nm respectively); a thermoelectrically cooled Andor DU401A-BV CCD camera; and a 60x water-immersion objective from Nikon (CFI PLAN APO IR 60xWI, MRD07650, NA = 1.25). In addition, an Olympus HXP 120 V light source is used together with several fluorescence filter sets to perform epi-fluorescence imaging [ET-DAPI (DC/49000), ET-CY3/TRITC (DC/49004) and ET-mCherry/Texas Red (DC/49008), Chroma Technology GmBH]. Power levels are monitored using a power meter inserted into the imaging path (PM100D, Thorlabs). A Digital Pixel Imaging System for temperature (37 degrees), CO_2_ level (5%) and humidity control has been installed around the microscope (Supplementary Fig. 5B).

The microscopy system, including lasers and CCD camera, were switched on 30 minutes prior to acquiring Raman data to allow for temperature stabilization. The Raman spectral resolution, dispersed by a 300 mm focal length spectrometer incorporating a 600 g/mm grating, varies between 3 and 5 cm^−1^. The spectrometer was calibrated using an HG-1 Mercury Argon calibration source (Hg and Ar Lines from 253–922 nm, Ocean Optics). Raman peak positions were calibrated using a silicon wafer sample before each measurement by adjusting the focus and the alignment of the fibre optic illumination delivery to maximise the signal counts in the first order Si Raman band at 520.7 cm^−1^. A value of 5000 cts (1^st^ order Si band at 785 nm, 214 mW, 0.05 s) was considered acceptable for performing Raman spectroscopy studies in cells in our instrument. Raman data acquisition from live cells was performed with the microscope in inverted configuration using either 488 nm or 785 nm excitation and WITec Control 4.1 software for hardware control. Coarse focus was achieved using a bright field imaging channel to minimize laser illumination; precise focus was then performed by acquiring Raman spectra from the sample using a low level of laser illumination, aiming to maximize the intensity of cellular Raman peaks while minimizing the intensity of contributions from the substrate. Line scans were acquired with 2.5–4.0 μm step size and area scans with 0.5 μm step size. Bright field images were acquired both before and after the Raman data acquisition process to provide a qualitative check on the health of the cells.

Data processing was performed using WITec Project Plus 4.1. Raw spectra (Supplementary Fig. 5C) were subjected to: cosmic ray removal (Supplementary Fig. 5D); baseline subtraction (Supplementary Fig. 5E; polynomial, order 5); and Savitzky-Golay smoothing (Supplementary Fig. 5F; width 11, order 3). For comparison of the qualitative changes in intensity of Raman bands under different conditions, we also performed normalization to the area under the curve (Supplementary Fig. 5G). Statistical significance was tested using Origin and for multivariate analysis, k-means clustering^42^ was performed in WITec Project Plus 4.1 while Principal Components Analysis and Partial Least Squares Discriminant Analysis were performed using the MATLAB PLS_Toolbox. For PLS-DA we performed additional pre-processing according to the method previously describe by Almeida *et al*. (preprocessing PLS-DA mode: vector normalization, 2nd derivative, smoothing and mean center)^32^. The PLS-DA model was built using all single Raman spectra acquired from the line scan method. The spectra were split into calibration and validation sets by removing every fourth spectrum to form the validation set. Cross validation was performed using venetian blinds, 10 data splits and the model was built using 6 latent variables, chosen to set the calibration and cross validation classification error average at 5%.

### Cell lines and evaluation of culture conditions for microscopy

Human fetal lung MRC5 pd19 (ECACC) and human lung carcinoma A549 (ATCC) immortalized cell lines were investigated during protocol optimization and cell comparison. MRC5 cells were grown in MEM (Gibco, Life Technologies, 51200-046) supplemented with 10% fetal bovine serum (Gibco, Life Technologies, 16000-044) and 2 mM L-glutamine (Life Technologies, 25030-024). A549 cells were grown in DMEM/F-12 with L-glutamine (Gibco, Life Technologies, 11039-021) supplemented with 10% fetal bovine serum (Gibco, Life Technologies, 16000-044). Cell lines were confirmed to be free of mycoplasma contamination using MycoProbe® Mycoplasma Detection Kit (R&D Systems).

Two primary human bronchial epithelial cells (HBECs) were commercially sourced (smoker, non-cancer, ATCC #PCS-300-010; never-smoker, non-cancer Lonza #CC-2540). Cells from ATCC and Lonza were received at passages six and seven, respectively. A third HBEC population was established directly from a patient sample by one of the authors (AH) and is referred to as PAP243 (never smoker, diagnosed with an atypical carcinoid, arising from neuroendocrine cells). PAP243 cells used for experimentation were received at passage three. PAP243 was established under Human Tissue Authority rec no.13/EE/0012). All HBECs were grown under serum-free conditions in Lonza BEGM BulletKit (CC-3170), at 37 °C and 5% CO_2_. HBECs were maintained at 65–80% confluence as the optimal conditions for these cultures. To prevent cellular differentiation, primary cells were not permitted to proliferate to more than 90% confluence.

To evaluate the optimal conditions for Raman micro-spectroscopy, several different substrates for cell seeding and culture media for cell analysis used in previous studies (example references given below) were directly compared under controlled conditions. These experiments were conducted using A549 cells due to the limited number of passages possible with MRC5 or primary cells. Raman spectra were recorded from: microscope cover glass (Marienfeld Cover glasses thickness No. 1.0 circular, dia. 25 mm, 0111650); quartz cover slips (UQG Optics, CFQ-2520); calcium fluoride slides (UV and IR grade, Crystran Limited) and a plastic cell culture dish traditionally used in live cell fluorescence microscopy (Greiner Bio-one, 627160). Review of the spectra at 488 nm (Supplementary Fig. 1A) and 785 nm (Supplementary Fig. 1B) confirmed that quartz and calcium fluoride provided the lowest background signals^21^. Spectra of standard solutions used to maintain live cells in microscopy were also tested at 488 nm (Supplementary Fig. 1C) and 785 nm (Supplementary Fig. 1D) including: standard cell culture media, in this case DMEM/F-12 without phenol red (tested both with and without serum); and salt solutions, in this case Hanks’ Balanced Salt Solution with 5.5 mM glucose (Gibco, Life Technologies, 14025-050), Live Cell Imaging Solution with 17.5 mM glucose (LCIS, ThermoFisher, A14291DJ), and Phosphate Buffered Saline (PBS, Life Technologies, 10010-056) with 17.5 mM glucose (Sigma, G8270). Cell proliferation in each of these cell culture media was evaluated in 96 well plates using automated phase contrast imaging (Supplementary Fig. 1E; Incucyte, Essen Biosciences), which together with the background spectra suggested that the standard complete medium with serum provided the best compromise for the health of the cells and the acquisition of spectra. All further experiments were thus performed in the recommended cell culture conditions for each cell line.

### Cell preparation for Raman micro-spectroscopy studies

Cells were maintained at 37 °C in humidified atmosphere containing 5% CO_2_. Cells for micro-spectroscopy analysis were seeded 12 hours prior to analysis in a 6 well plate with a 25 mm round quartz coverslip (UQG Optics, CFQ-2520) at a density of 2 × 10^5^ A549 cells or 1 × 10^5^ MRC5 cells per well and incubated with phenol red-free medium with serum (vendor as above). Immediately prior to Raman micro-spectroscopy measurements, coverslips were mounted into the Attofluor cell chamber (Invitrogen, cat. no. A-7816). Cells were washed with PBS to remove any unattached cells and fresh complete medium was added. Primary HBEC cells were seeded at 2 × 10^5^ cells per well and incubated with Lonza BEGM BulletKit media for 12 hours prior to analysis. A total of 30 cells (biological replicates) were studied for each bronchial cell culture, with 10 cells studied per experiment and 3 replicate experiments performed for each cell line.

Comparison between label-free Raman imaging and specific staining of intracellular compartments using fluorescent dyes and antibodies can be achieved with appropriate hardware and cells staining protocols. Previous authors have achieved this using a range of dyes, for example: an alpha-actin for myofibrils^43^; DAPI for cell nuclei^43^; Oil Red O for lipid droplets^44^; anti-COX-IV for mitochondria^10^; anti-Calnexin for endoplasmic reticulum^10^; and anti-Syntaxin-6-antibody to denote the Golgi apparatus^10^. Following our Raman micro-spectroscopy experiments, live cell cultures within the Attofluor chamber were exposed to NucBlue at 1 drop per 1 mL culture media (Invitrogen, R37605 Hoechst 33342) and Nile Red (Acros Organics, 7385-67-3) in order to assist with identification of nuclear and lipid regions in our Raman area-scan data. Nile Red was prepared by adding 1 μL of filtered (pore size 200 μm) 0.5 mM Nile Red dissolved in 60% isopropanol/dH_2_O to 1 mL culture media. NucBlue and Nile Red stains were applied to the sample at the same time and incubated for 10 min. Samples were then washed twice with PBS before fresh warm phenol red-free medium with serum was applied for epi-fluorescence imaging on the same microscope.

## Electronic supplementary material

Combined supplementary information file
