# Supplementary material for: Raman micro-spectroscopy for accurate identification of primary human bronchial epithelial cells
Source: Sci Rep. 2018 Aug 22;8:12604. doi: 10.1038/s41598-018-30407-8 (PMC6105656; doi:10.1038/s41598-018-30407-8)
Supplement: Supplementary file 1 — Combined supplementary information file [file 41598_2018_30407_MOESM1_ESM.pdf]

Supplementary Information for:

**Raman micro-spectroscopy for accurate identification of primary human bronchial epithelial cells.**

Jakub M Surmacki[1,2], Benjamin J Woodhams[1,2], Alexandria Haslehurst[2], Bruce AJ Ponder[2] and Sarah E Bohndiek[1,2,*]

[1] Department of Physics, University of Cambridge. Cavendish Laboratory, JJ Thomson Avenue, Cambridge, CB3 0HE, United Kingdom

[2] Cancer Research UK Cambridge Institute. University of Cambridge. Li Ka Shing Centre, Robinson Way, Cambridge CB2 0RE, United Kingdom

## Supplementary Figures:

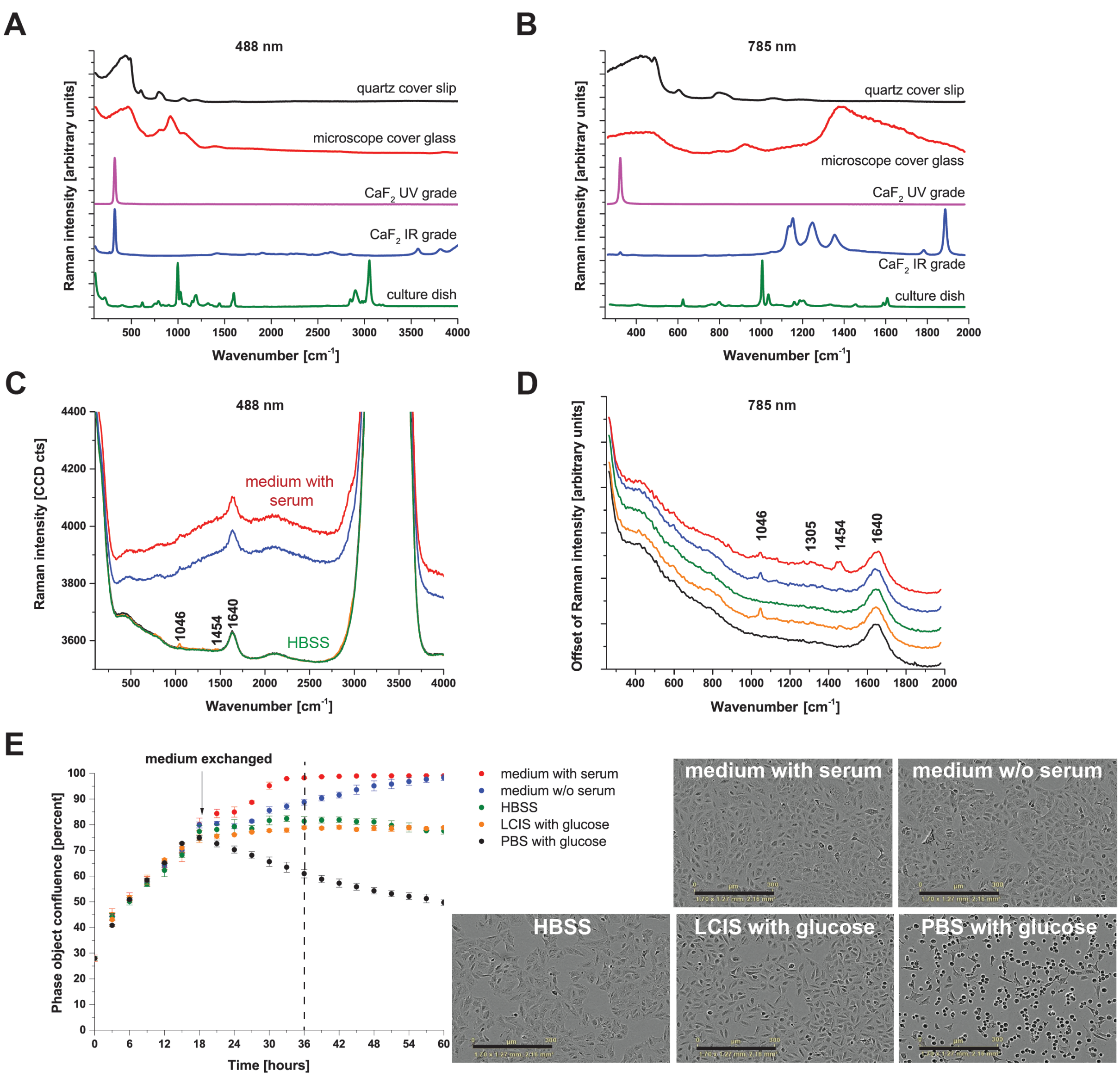


**Supplementary Figure 1. Evaluation of different live cell substrates and culture media / solutions**. Raman spectra taken at 488 nm (A) and 785 nm (B) of substrates including: a quartz cover slip; microscope cover glass; calcium fluoride $CaF_2$ (UV and IR grade) and a plastic culture dish. Raman spectra taken at 488 nm (C) and 785 nm (D) of culture media / solutions including: DMEM/F-12 medium without phenol red (with and without serum, red and blue respectively); Hanks' Balanced Salt Solution (HBSS, green); Live Cell Imaging Solution (LCIS, yellow) with glucose; and phosphate

buffered saline (PBS, black) with glucose. All Raman spectra were acquired with the following characteristics: 488 nm: 10 mW, with 0.5 s exposure time and 100 accumulations. 785 nm: 150 mW, with 1.0 s exposure time and 100 accumulations. (E) Automated phase contrast imaging of A549 cells to evaluate the influence of the cell culture media / solutions presented in (C,D) on cellular proliferation (microscope images present at 36 hours from each solution tested, black scale bar 300 $\mu$m).

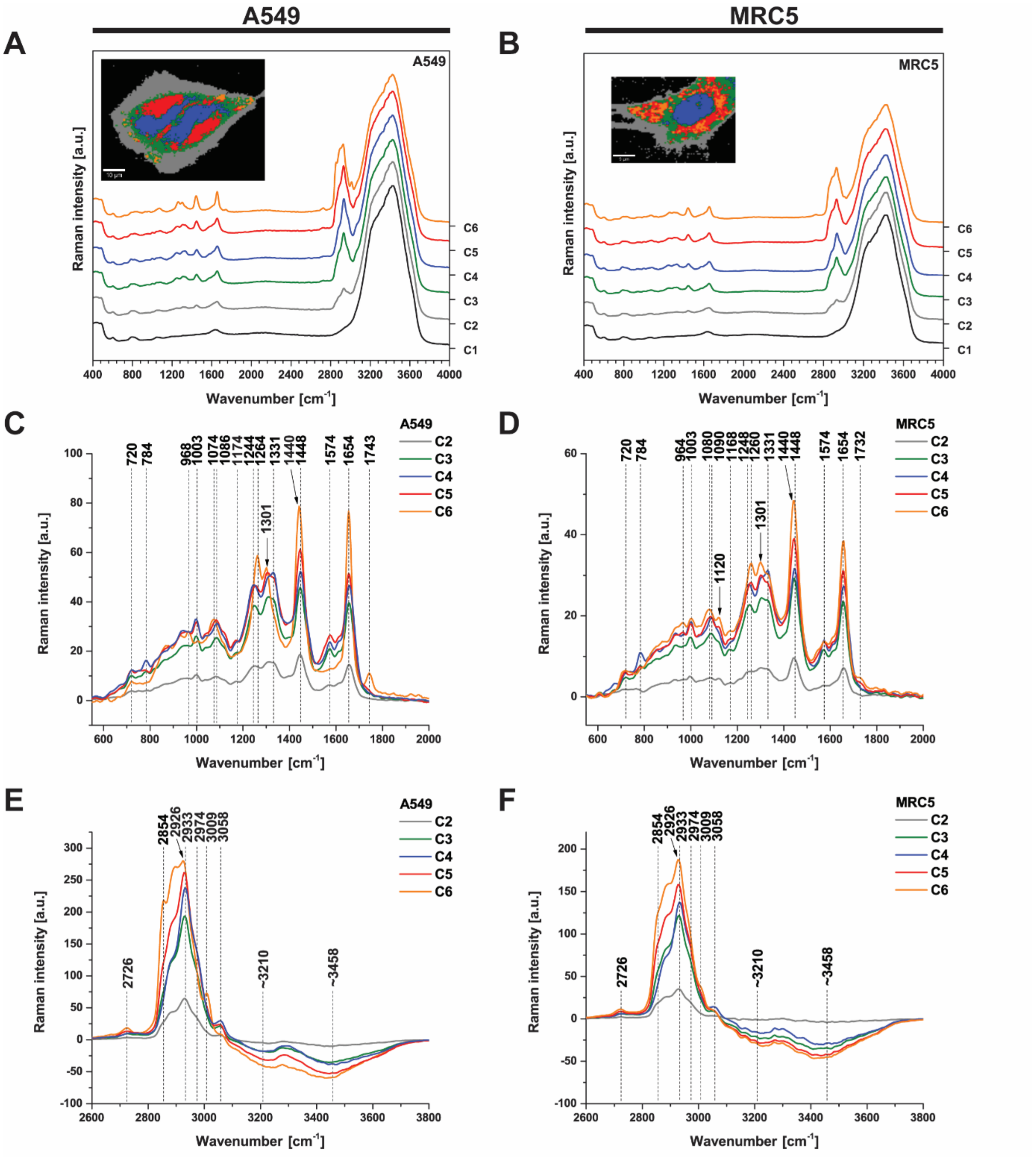


**Supplementary Figure 2. Cluster analysis of the representative A549 and MRC5 Raman cell maps.** Maps were presented in Figure 1 of the main manuscript. We performed the following assignments of clusters based on spectra presented here: Black (cluster 1) = area without the cells (background); Grey (cluster 2) = cell border; Green (cluster 3) = cytoplasm; Blue (cluster 4) = nucleic acids within the nucleus (main

contributions at 784, 1086-1090, 1574 and 2974 $cm^{-1}$), corresponding to areas denoted in the fluorescence images; Red (cluster 5) = endoplasmic reticulum/mitochondria (main contribution at 2930 $cm^{-1}$); Orange (cluster 6) = lipid droplets (main contributions of unsaturated lipids at 2854 and 3009 $cm^{-1}$ [1–4]), again corresponding to areas denoted in the fluorescence images. (C-F) Raman clusters spectra after background (cluster 1) removed.

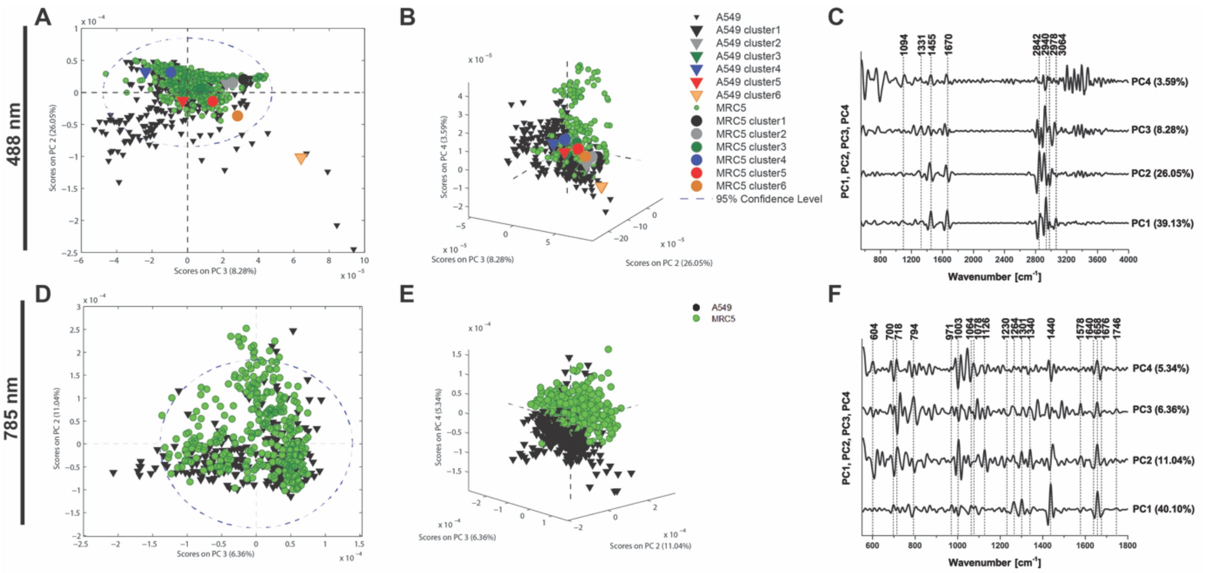


**Supplementary Figure 3. Principal component analysis of the second derivative of all Raman spectroscopy data from A549 and MRC5 cells at 488 nm and 785 nm.** Scatter plots are shown of the scores of each single Raman spectrum for the second and third principal components from the A549 (black triangles) and MRC5 cells (green circles) at 488 nm (A,B) and 785 nm (D,E). Large symbols illustrate the mapping of the cluster spectra derived from the area scan onto these principal components (Supplementary Fig. 3A,B) as follows: C1 – background (black), C2 – cell border (gray), C3 – cytoplasm (green), C4 – nucleus (blue), C5 – endoplasmic reticulum /mitochondria (red), C6 – lipid droplets (orange). (C,F) Loadings plot of PC1, PC2, PC3 and PC4, indicating the peaks that contribute to these principal components derived at 488 nm and 785 nm respectively. Preprocessing PCA mode: normalization to area, 2$^{nd}$ derivative, mean center.

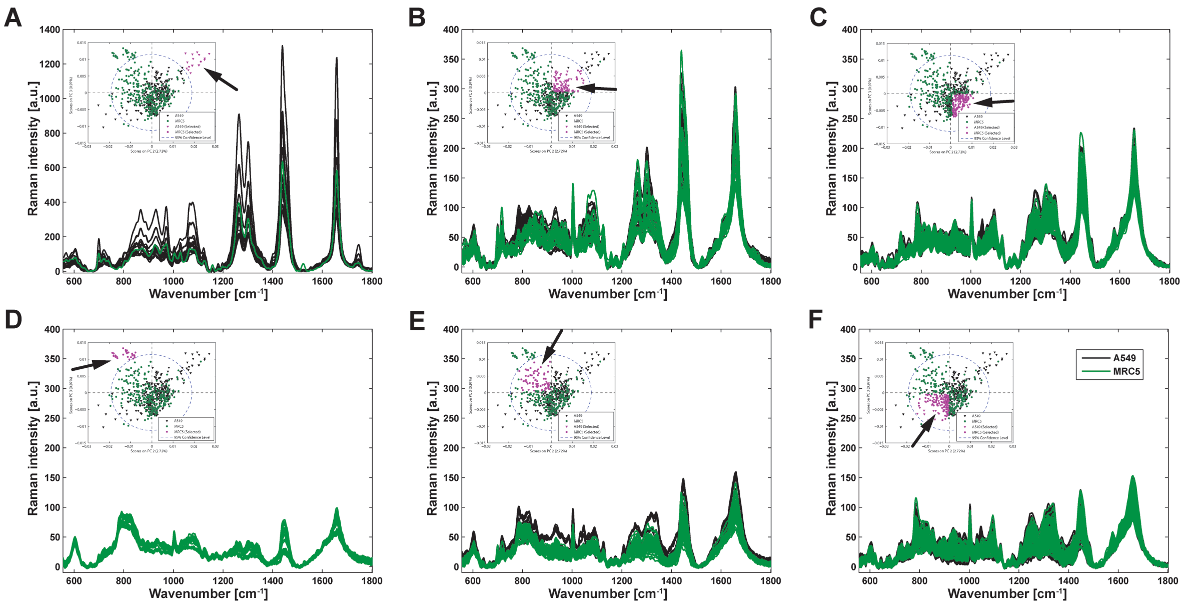


**Supplementary Figure 4. Raman spectra from specific regions of the principal components analysis in Figure 6.** Raman spectra of the A549 (black line) and MRC5 (green line) cells at 785 nm (A-F) computed from the groups of points highlighted in violet. Inset graphs are the scatterplots of the score values of each single Raman spectrum for the second and third principal components from the A549 (black triangles) and MRC5 cells (green circles).

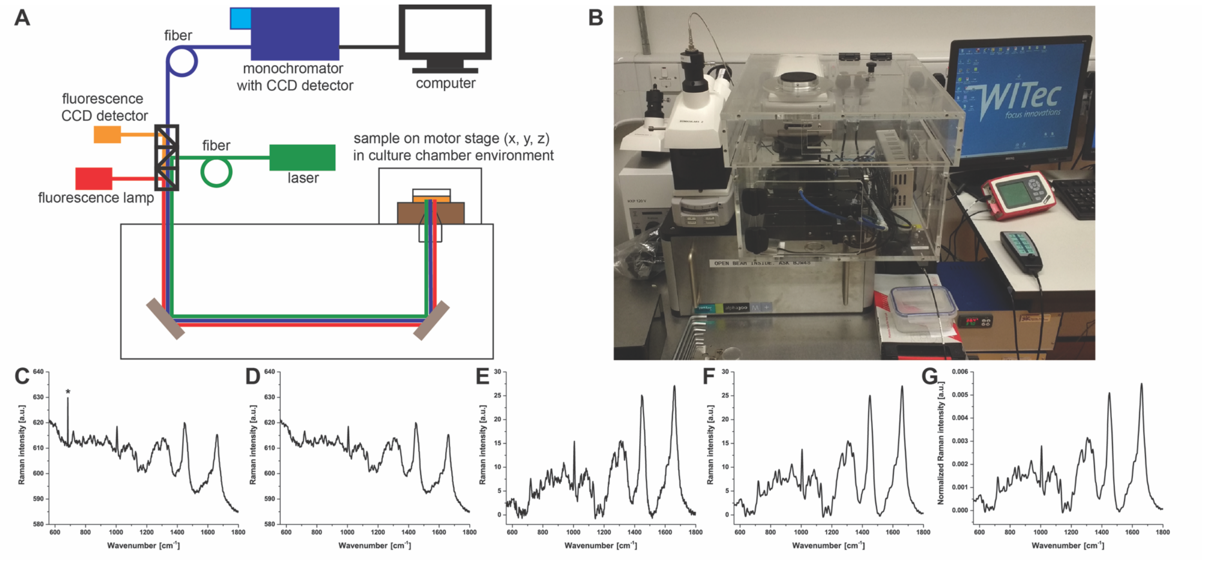


**Supplementary Figure 5: Acquisition and processing of confocal Raman micro-spectroscopy data from live cells.** (A) Schematic of the instrument used for confocal Raman and epi-fluorescence microscopy. (B) Photo of the live cell Raman microscope. Illustration of the pipeline for data analysis, proceeding from the raw data (C) through the following steps: cosmic ray removal (D); baseline subtraction (polynomial, order 5) (E); Savitzky-Golay smoothing (width 11, order 3) (F); and normalization (optional) to the area under the curve (G).

**Supplementary Table 1. Assignment of Raman bands observed in the cells.** Bands highlighted in bold were most discriminatory for the human cell lines examined using PLS-DA according to the variable importance in projection scores in Figure 6.

| Peak position [$cm^{-1}$] | Mode description[5,6,7,8,9,1,10,11,12,13] |
|---|---|
| **540-600** | **Out of plane C=O bending, amide IV, proteins** |
| **625-770** | **OCN bending, amide IV, proteins** |
| **640-800** | **NH bending, amide V, proteins** |
| 720 | C-$H_2$ deformation, $N^+$-$(CH_3)_3$ symmetric stretching, lipids |
| 784 | Cytosine, uracil, thymine, pyrimidine bases, ring breathing modes, DNA |
| **828** | **O-P-O stretching, DNA/RNA** |
| **879-881** | **Tryptophan, proteins, carbohydrates** |
| **940-944** | **Skeletal modes, polysaccharides/carbohydrates** |
| 964-968 | $CH_2$ wagging |
| **998-1006** | **Phenylalanine, proline, symmetric stretching (ring breathing) mode of phenyl group** |
| **1043-1049** | **Proline, collagen, proteins, carbohydates** |
| 1074-1086 | C-C stretching, C-O stretching, phospholipids, proteins, carbohydrates |
| 1088-1095 | $PO_2^-$ , symmetric stretching mode of phosphate esters, DNA/RNA |
| **1120** | **C-C stretching, C-O stretching, saccharides/lipids** |

| | |
|---|---|
| **1168-1174** | **$C-C_6H_5$ phenylalanine, tryptophan** |
| **1230-1350** | **Extended amide III, coupled C-H, N-H deformation modes, peptide backbone** |
| 1260-1264 | =CH deformation, lipids |
| **1298-1301** | **$CH_2$ twist, lipids** |
| **1315-1320** | **Guanine, nucleic acids** |
| 1331 | Adenine/guanine, nucleic acids |
| **1440-1450** | **$CH_2$ and $CH_3$ deformations, antisymmetric methyl and methylene deformations, phospholipids** |
| **1480-1580** | **C-N stretching and N-H bending, amide II, proteins** |
| 1518 | C=C stretching |
| 1574 | C=C stretching, purine bases, DNA/RNA |
| 1654-1660 | C=C stretching, lipids |
| **1600-1690** | **Amide I C=O stretching mode, peptide linkage** |
| 1740-1746 | C=O stretching, ester group of lipids and phospholipids |

**Supplementary Table 2: Results of the Mann-Whitney Test (300 spectra from 30 cells per group).** Statistical significance was tested using the nonparametric Mann-Whitney test as our populations were not normally distributed. Spectra normalized to the area under curve, excitation 785 nm * indicates p<0.05.

| Raman peak position [$cm^{-1}$] | Cell line | Median | Q1 | Q3 | p-value |
|---|---|---|---|---|---|
| 784 | A549 | 0.00209 | 0.00163 | 0.00241 | 4.40E-15 * |
| | MRC5 | 0.00257 | 0.00180 | 0.00257 | |
| 1003 | A549 | 0.00259 | 0.00233 | 0.00279 | 0.911 |
| | MRC5 | 0.00258 | 0.00234 | 0.00281 | |
| 1264 | A549 | 0.00237 | 0.00216 | 0.00258 | 3.11E-13 * |
| | MRC5 | 0.00214 | 0.00174 | 0.00245 | |
| 1301 | A549 | 0.00268 | 0.00238 | 0.00299 | 6.00E-15 * |
| | MRC5 | 0.00238 | 0.00182 | 0.00276 | |
| 1440 | A549 | 0.00407 | 0.00318 | 0.00479 | 2.20E-07 * |
| | MRC5 | 0.00339 | 0.00295 | 0.00439 | |
| 1658 | A549 | 0.00513 | 0.00480 | 0.00551 | 0.531 |
| | MRC5 | 0.00517 | 0.00480 | 0.00549 | |

**Supplementary Table 3: Confusion matrix from PLS-DA analysis of all individual line-scan spectra.**

| | | | | | |
|---|---|---|---|---|---|
| **Model results** | | | | | |
| | A549 | ATCC | LONZA | MRC5 | PAP243 |
| Predicted as A549 | 206 | 0 | 1 | 29 | 0 |
| Predicted as ATCC | 0 | 420 | 22 | 0 | 7 |
| Predicted as LONZA | 0 | 20 | 390 | 1 | 21 |
| Predicted as MRC5 | 18 | 6 | 2 | 194 | 4 |
| Predicted as PAP243 | 1 | 4 | 35 | 1 | 418 |
| **CV results** | | | | | |
| | A549 | ATCC | LONZA | MRC5 | PAP243 |
| Predicted as A549 | 204 | 0 | 1 | 29 | 0 |
| Predicted as ATCC | 0 | 417 | 26 | 0 | 7 |
| Predicted as LONZA | 0 | 23 | 386 | 0 | 22 |
| Predicted as MRC5 | 20 | 6 | 3 | 195 | 4 |
| Predicted as PAP243 | 1 | 4 | 34 | 1 | 417 |
| **Prediction results** | | | | | |
| | A549 | ATCC | LONZA | MRC5 | PAP243 |
| Predicted as A549 | 68 | 0 | 0 | 11 | 0 |
| Predicted as ATCC | 0 | 137 | 4 | 0 | 0 |
| Predicted as LONZA | 1 | 6 | 132 | 0 | 7 |
| Predicted as MRC5 | 6 | 3 | 1 | 63 | 1 |
| Predicted as PAP243 | 0 | 4 | 13 | 1 | 142 |

**Supplementary Table 4: Confusion matrix from PLS-DA analysis of all averaged line-scan spectra.**

| **Model results** | | | | | |
|---|---|---|---|---|---|
| | A549 | ATCC | LONZA | MRC5 | PAP243 |
| Predicted as A549 | 22 | 0 | 0 | 0 | 0 |
| Predicted as ATCC | 0 | 44 | 1 | 0 | 0 |
| Predicted as LONZA | 0 | 0 | 41 | 0 | 0 |
| Predicted as MRC5 | 0 | 0 | 0 | 22 | 0 |
| Predicted as PAP243 | 0 | 0 | 2 | 0 | 44 |
| **CV results** | | | | | |
| | A549 | ATCC | LONZA | MRC5 | PAP243 |
| Predicted as A549 | 21 | 0 | 0 | 0 | 0 |
| Predicted as ATCC | 0 | 43 | 2 | 0 | 0 |
| Predicted as LONZA | 0 | 1 | 40 | 0 | 0 |
| Predicted as MRC5 | 1 | 0 | 0 | 22 | 0 |
| Predicted as PAP243 | 0 | 0 | 2 | 0 | 44 |
| **Prediction results** | | | | | |
| | A549 | ATCC | LONZA | MRC5 | PAP243 |
| Predicted as A549 | 7 | 0 | 0 | 0 | 0 |
| Predicted as ATCC | 0 | 15 | 0 | 0 | 0 |
| Predicted as LONZA | 0 | 1 | 15 | 0 | 2 |
| Predicted as MRC5 | 1 | 0 | 0 | 8 | 0 |
| Predicted as PAP243 | 0 | 0 | 1 | 0 | 14 |

**References:**


1. Abramczyk, H. *et al.* The role of lipid droplets and adipocytes in cancer. Raman imaging of cell cultures: MCF10A, MCF7, and MDA-MB-231 compared to adipocytes in cancerous human breast tissue. *Analyst* **140,** 2224–2235 (2015).
2. Surmacki, J., Brozek-Pluska, B., Kordek, R. & Abramczyk, H. The lipid-reactive oxygen species phenotype of breast cancer. Raman spectroscopy and mapping, PCA and PLSDA for invasive ductal carcinoma and invasive lobular carcinoma. Molecular tumorigenic mechanisms beyond Warburg effect. *Analyst* **140,** 2121–33 (2015).
3. Brozek-Pluska, B., Kopec, M., Surmacki, J. & Abramczyk, H. Raman microspectroscopy of noncancerous and cancerous human breast tissues. Identification and phase transitions of linoleic and oleic acids by Raman low-temperature studies. *Analyst* **140,** 2134–43 (2015).
4. Surmacki, J., Musial, J., Kordek, R. & Abramczyk, H. Raman imaging at biological interfaces: applications in breast cancer diagnosis. *Mol Cancer* **12,** 48 (2013).
5. Schie, I. & Huser, T. Methods and applications of Raman microspectroscopy to single-cell analysis. *Appl. Spectrosc.* **67,** 813–28 (2013).
6. Boyd, A. R., McManus, L. L., Burke, G. A. & Meenan, B. J. Raman spectroscopy of primary bovine aortic endothelial cells: A comparison of single cell and cell cluster analysis. *J. Mater. Sci. Mater. Med.* **22,** 1923–1930 (2011).
7. Swain, R. J., Jell, G. & Stevens, M. M. Non-invasive analysis of cell cycle dynamics in single living cells with Raman micro-spectroscopy. *J. Cell. Biochem.* **104,** 1427–1438 (2008).

8. Notingher, I. *et al.* In situ characterisation of living cells by Raman spectroscopy. *Spectroscopy* **16,** 43–51 (2002).

9. Notingher, I., Verrier, S., Haque, S., Polak, J. M. & Hench, L. L. Spectroscopic study of human lung epithelial cells (A549) in culture: living cells versus dead cells. *Biopolymers* **72,** 230–40 (2003).

10. Stone, N., Kendall, C., Smith, J., Crow, P. & Barr, H. Raman Spectroscopy for Identification of Epithelial Cancers. *Faraday Discuss.* **126,** 141–157 (2004).

11. Surmacki, J., Musial, J., Kordek, R. & Abramczyk, H. Raman imaging at biological interfaces: applications in breast cancer diagnosis. *Mol. Cancer* **12,** 48 (2013).

12. Notingher, I., Bisson, I., Polak, J. M. & Hench, L. L. In situ spectroscopic study of nucleic acids in differentiating embryonic stem cells. *Vib. Spectrosc.* **35,** 199–203 (2004).

13. De Gelder, J., De Gussem, K., Vandenabeele, P. & Moens, L. Reference database of Raman spectra of biological molecules. *J. Raman Spectrosc.* **38,** 1133–1147 (2007).
